# Hydrogen Cyanide in the Rhizosphere: Not Suppressing Plant Pathogens, but Rather Regulating Availability of Phosphate

**DOI:** 10.3389/fmicb.2016.01785

**Published:** 2016-11-18

**Authors:** Tomaž Rijavec, Aleš Lapanje

**Affiliations:** ^1^Institute of Metagenomics and Microbial TechnologiesLjubljana, Slovenia; ^2^Department of Environmental Sciences, Jožef Stefan InstituteLjubljana, Slovenia; ^3^Remote Controlled Theranostic Systems Lab, Saratov State UniversitySaratov, Russia

**Keywords:** cyanide, rhizobacteria, *Pseudomonas*, antimicrobial, PGP, phosphate availability

## Abstract

Plant growth promoting rhizobacteria produce chemical compounds with different benefits for the plant. Among them, HCN is recognized as a biocontrol agent, based on its ascribed toxicity against plant pathogens. Based on several past studies questioning the validity of this hypothesis, we have re-addressed the issue by designing a new set of *in vitro* experiments, to test if HCN-producing rhizobacteria could inhibit the growth of phytopathogens. The level of HCN produced by the rhizobacteria *in vitro* does not correlate with the observed biocontrol effects, thus disproving the biocontrol hypothesis. We developed a new concept, in which HCN does not act as a biocontrol agent, but rather is involved in geochemical processes in the substrate (e.g., chelation of metals), indirectly increasing the availability of phosphate. Since this scenario can be important for the pioneer plants living in oligotrophic alpine environments, we inoculated HCN producing bacteria into sterile mineral sand together with germinating plants and showed that the growth of the pioneer plant French sorrel was increased on granite-based substrate. No such effect could be observed for maize, where plantlets depend on the nutrients stored in the endosperm. To support our concept, we used KCN and mineral sand and showed that mineral mobilization and phosphate release could be caused by cyanide *in vitro*. We propose that in oligotrophic alpine environments, and possibly elsewhere, the main contribution of HCN is in the sequestration of metals and the consequential indirect increase of nutrient availability, which is beneficial for the rhizobacteria and their plant hosts.

## Introduction

A beneficial rhizobacterial community is essential for growth and development of the plant host (Morgan et al., 2005) and includes plant growth promoting (PGP) rhizobacteria. These bacteria regulate plant developmental processes, inhibit the growth of phytopathogens, increase nutrient availability and fix nitrogen (reviewed in Lugtenberg and Kamilova, 2009). In this context, the association between *Pseudomonas* spp. and the plant rhizosphere has been well-documented (Dutta and Podile, 2010) and bacteria from this genus are currently used as model organisms for studies on root colonization (Lugtenberg et al., 2001). To promote plant growth, *Pseudomonas* spp. bacteria colonize competitor niches, produce iron-chelating and antibiotic compounds, and excrete volatiles, which induce plant systemic resistance (reviewed in Santoyo et al., 2012). Fluorescent pseudomonads, in particular, have been extensively studied and most often implicated in biocontrol of plant pathogens, due to their ability to produce several antimicrobial compounds, including HCN (reviewed in Haas and Défago, 2005).

One of the initial studies on HCN-producing pseudomonads (HPP), which concluded that HCN could be toxic for plant pathogens (Voisard et al., 1989), was based on experiments that did not unambiguously exclude the activity of other antimicrobial compounds produced by these bacteria. Pal et al. (2000) much later concluded that HCN was an unlikely biocontrol agent and that bacterial products, like pigments and antibiotics, were much more effective against fungal pathogens. Since HCN also has no specific action against pathogenic microorganisms and even caused phytotoxic effects in most *in vitro* experiments (Alström and Burns, 1989; Kremer and Souissi, 2001; Rudrappa et al., 2008; Blom et al., 2011), this cast further doubt on the proposed antimicrobial action of HCN. To address the inconsistencies of these past observations, we re-examined the role of HCN in biocontrol, by determining *in vitro* whether the amount of HCN produced by the isolated HPP strains could be correlated with their biocontrol activity. In our newly designed experiments we: (*i*) tested the level of HCN production by rhizospheric isolates in optimal *in vitro* conditions, the strain's potential to produce HCN, and their antimicrobial activity against phytopathogenic bacteria and fungi *in vitro*, seeking a correlation and (*ii*) examined whether KCN inhibits the growth in HCN-producing (HCN+) and non-producing (HCN−) rhizospheric isolates.

HCN produced by the rhizobacteria could also act in other ways. Based on its ability to form complexes with transitional metals in the mineral substrate (Faramarzi and Brandl, 2006; Fairbrother et al., 2009), we developed the hypothesis in which the ecological function of HCN is based on its interference with the release of elements from the mineral substrate. Recent studies on mineral weathering in natural environments (Frey et al., 2010; Lapanje et al., 2012; Wongfun et al., 2014) indicated that HCN producing bacteria (HPB) promoted the mobilization of elements from rock forming minerals. We reasoned that by affecting the solubility of these elements, HCN can indirectly also interfere with phosphorus availability. The latter is particularly problematic in different types of soils (reviewed in Hinsinger, 2001) as complexation of phosphate by calcium in basic soils) or by aluminum and iron in acidic soils (Sanyal and De Datta, 1991) leads to the formation of insoluble metal-phosphate complexes (Chabot et al., 1996). Since in acidic soils, HCN can interact with iron, the main contribution of biogenic HCN is not in the increased weathering of iron, but in its sequestration, leading to increased availability of phosphate. We hypothesized, that biogenic HCN could increase availability of nutrients in the substrate, resulting in increased PGP. When testing this hypothesis, it is important to keep in mind that the effects of HCN can be masked by other PGP properties, which also increase nutrient availability. This could include the characteristics of different types of bedrock (mineral composition and pH), HCN-unrelated antimicrobial activity of bacteria and physiological characteristics of host plants. For example, the screening of rhizobacteria for the production of compounds associated with PGP (Cattelan et al., 1999; Ahmad et al., 2008) has shown that single strains can have multiple PGP activities. Their effect is nevertheless specific and no two systems differing in soil type, plant species or combination of rhizobacteria in the community will show identical promotion of plant growth (Cakmakçi et al., 2006; Egamberdiyeva, 2007). A single bacterial strain will not show the same positive effect in two different environments and strains that will not test positive for any PGP trait *in vitro* might still promote plant growth or *vice versa* (Cattelan et al., 1999).

For these reasons, we can expect that two completely different plant types, crop plant (e.g., maize) and alpine pioneer plant (e.g., French sorrel), will show different growth characteristics stemming from the differences in their ecological adaptation. At the same time however, they will not benefit equally from HPB when grown on different substrates, like limestone or granite mineral soil, because: (*i*) these substrates differ in pH and chemical composition (N, K, P content), (*ii*) plant-microbe interactions differ between plant type and developmental stage (Chaparro et al., 2014), and (*iii*) plants with small seed size are being compared to plants with large storage tissues, more dependent on obtaining nutrients from the rhizosphere (reviewed in Harley and Gilkes, 2000).

Taking into account all the aforementioned facts, the second aim of our study was to test whether biogenic HCN rather than antimicrobial action, can actually be linked more to increased availability of nutrients. We tested our hypothesis on the role of HCN in increased nutrient availability by (*i*) determining the effect of HCN producers on the growth of two different types of plants inoculated onto two types of soil substrates and by *in vitro* experiments, where we determined (*ii*) how HCN levels increased solubilization of granite mineral substrate and availability of phosphate and (*iii*) how biologically relevant concentrations of cyanide interact with Fe^3+^ and ${\text{PO}}_{4}^{3-}$ ions.

## Methods

### Isolation of bacterial strains and collection of bedrock material

The material for the isolation of bacterial strains, rhizosphere soil, and plant-unassociated mineral soil, was collected on two alpine sites, one granite-based (Damma glacier forefront, Switzerland, 2.100 m a.s.l., 46°38′10.6″N 8°27′40.6″E) and the other a limestone-based site (mountain scree, Lake Krn, Slovenia, 1.400 m a.s.l., 46°16′57.4″N 13°40′53.9″E). Bacteria were isolated by washing the material (1–5 g) in sterile 0.9% NaCl (Sigma, USA) and diluting the obtained solution 10-fold (down to 10^−9^). A total of 100 μL of the extract was spread on King's B agar plates (KB) (20 g L^−1^ peptone, 1.5 g L^−1^ K_2_HPO_4_, MgSO_4_·7H_2_O, 10 mL L^−1^ glycerol, 15 g L^−1^ agar) and the plates were incubated at 25°C for 14 days. From these primary plates *Pseudomonas*-like colony phenotypes (slimy or producing diffusible fluorescent pigment) were isolated. Pure cultures were stored at −80°C prior to further analysis. In plant growth experiments, samples of raw granite and limestone bedrock samples (granite rocks from Damma glacier [46°38′ 9.96″N, 8°27′39.6″E] fore-field and limestone rocks from limestone gravel tank, Ljubljana, Slovenia [46°04′33.0″N 14°32′56.8″E]) were used. Raw bedrock material was ground to give a particle size <1 mm (according to ISO 3310), soaked in distilled water overnight, washed thoroughly with distilled water, dried and autoclaved before further experiments.

### Quantitative measurements of HCN production by rhizobacterial isolates

The concentration of HCN produced by the isolates was determined for each strain in overnight liquid cultures. The quantitative method used to measure extracellular and dissolved free non-complexed cyanide ions (CN^−^) in the liquid medium was based on a modified colorimetric methemoglobin method (von Rohr et al., 2009), initially described by Baumeister and Schievelbein (1971). Overnight bacterial cultures were prepared in Luria-Bertani broth (Miller) (LB) (Sigma-Aldrich, USA) supplied with 5 g L^−1^ glycine (Sigma-Aldrich, USA) (LB_gly_) and the pH was adjusted to 7.4. This medium stimulates the production of HCN thus enabling the determination of the maximum potential for HCN production. The methemoglobin reagent was prepared as follows: 0.34% (w/v) hemoglobin (Sigma-Aldrich, USA) was dissolved in 4 mM NaNO_2_ (Sigma-Aldrich, USA) and incubated for 10 min, phosphate buffer (137 mM NaCl, 2.7 mM KCl, 10 mM Na_2_HPO_4_, 1.8 mM KH_2_PO_4_) was added in a 1:1 ratio and the solution was mixed and incubated for a further 30 min at room temperature. A KCN (Alfa Aesar, Germany) solution was used as the concentration standard. All liquid samples were prepared and spectrophotometric measurements at 424 nm were carried out using a SynergyH4 multi-plate reader (Biotek, USA) and clear bottom 96 well micro assay plates (Greiner Bio-One, Germany) based on the method described by von Rohr et al. (2009). In biocontrol experiments, HCN production by rhizobacterial isolates was monitored using semi-quantitative rapid detection of HCN production according to Castric and Castric (1983) (Figure S1).

### Screening of bacterial strains for weathering-related substances

Bacterial strains were screened for production of substances promoting iron acquisition (siderophores) and solubilization of inorganic phosphate (indicator or organic acid production). Pyoverdine production was determined by growing the strains on a solid KB medium at room temperature for up to 5 days then placing the plate under UV light (254 nm) using a Biomertra Ti5 transilluminator (Biometra, Germany). A positive signal was observed as the green fluorescent light emitting from the colony and the surrounding medium, while the negative signal was observed as the absence of any fluorescent light in the colony or the medium (Hamdan et al., 1991). Production of other siderophores was assessed by a technically modified CAS assay (Schwyn and Neilands, 1987). Strains were grown on LB agar (Sigma, USA), poured on one half of the plate, and siderophore production was examined on the CAS indicator medium, applied to the other half of the plate. The production of released siderophores was monitored visually according to Pérez-Miranda et al. (2007) on the indicator half of the plate after the siderophores diffused away from the colonies growing on the LB agar side of the plate. Inorganic phosphate solubilization ability was determined on Pikowskaya agar (0.5 g L^−1^ yeast extract, 10 g L^−1^ dextrose, 5 g L^−1^ Ca_3_(PO_4_)_2_, 0.5 g L^−1^ (NH_4_)_2_SO_4_, 0.2 g L^−1^ KCl, 0.1 g L^−1^ MgSO_4_, 0.0001 g L^−1^ MnSO_4_, 0.0001 g L^−1^ FeSO_4_) according to Pandey et al. (2006), by visible detection of a transparent zone around the colony.

### Inhibition of growth of phytopathogenic microorganisms

The newly designed experiments were set up to test the biocontrol activity of rhizospheric bacteria with different potential for HCN production (either positive or negative, as well as different strength of production). The biocontrol activities of the 11 HCN+ and 9 HCN− rhizobacterial strains against agriculturally important phytopathogenic fungi *Fusarium moniliforme* EXF1and *F. graminearum* EXF2 and phytopathogenic bacteria *Pseudomonas syringae* pv. *syringae* z1, *P. syringae* pv. *coronafaciens* z1238, *Erwinia carotovora* pv. *carotovora* z87 and *Xanthomonas campestris* pv. *campestris* z1352 were tested on solid LB agar medium (LBA) and LBA supplemented with glycine (5 g L^−1^, pH = 7.4; LBA_gly_) to promote HCN production *via* the glycine pathway (Castric, 1977). Two assay approaches were used to test the isolates' anti-fungal activity by either direct contact or no contact between the fungus and the bacterium, to determine if volatile compounds or compounds that diffused into the medium were the reason for the observed inhibition of growth of the fungi. In both cases, agar plugs (*d* = 5 mm) containing fungal hyphae were prepared by cutting out the plugs from solid potato dextrose agar (PDA) medium (potato starch 4 g L^−1^, glucose 20 g L^−1^, agar 15 g L^−1^) overgrown by pathogenic fungi. In the first approach, bacteria were spread evenly over the surface of LBA and LBA_*gly*_ media. Concurrently, four agar plugs, with fungal hyphae facing downwards, were aseptically placed on this previously inoculated medium surface. In the second approach, bacteria were streaked on the plate surface in a central line, dividing the agar plate in half and the agar plugs were placed on the surface on both sides of the line. Assay plates were incubated for 10 days at 25°C and fungal growth was monitored daily by measuring its growth radius. The growth of the fungi was compared to their growth in the control experiment, where only the plugs with fungal mycelium were plated on the media and no bacteria were present.

For antibacterial assays, rhizobacteria were cultivated in LB overnight at room temperature. Phytopathogenic bacteria were spread evenly over LBA and LBA_gly_ medium surface. Sterile paper discs (*d* = 5 mm) soaked in the liquid cultures of the rhizobacteria were placed on the solid medium surface. Agar plates were incubated for 10 days at 25°C and the formation of inhibition zones round the discs was monitored daily (Figure S2).

### Effect of HCN on the growth of rhizobacterial isolates

To determine whether HCN+ rhizobacterial isolates were more resistant to HCN in solution compared to HCN− isolates, the minimal inhibitory concentration (MIC) of cyanide (KCN) and the effect of cyanide on the growth of bacteria were analyzed using four HCN+ and four HCN− rhizobacterial isolates. A concentration series of 100 μM to 1 mM (100 μM increment) and 1 mM to 10 mM (1 mM increment) of KCN in LB was used to determine MIC. A total of 1 μL of an overnight culture was inoculated into the liquid medium and OD_600_ was measured at 24, 48, and 72 h. MIC was described as the lowest KCN concentration, where we did not observe any growth of the bacteria by measuring OD after 72 h incubation time. The effect of KCN of the growth of bacteria was examined after spiking the medium with KCN at final concentrations of 100 μM and 200 μM that correspond to the concentrations produced by HCN+ environmental isolates in liquid culture conditions. LB medium was supplemented with 100 μM (LB_100_) or 200 μM KCN (LB_200_), inoculated with 1 μL of overnight liquid culture and was monitored hourly (OD_600_) during incubation at 25°C. The pH was measured in the sterile fresh medium and the medium of stationary phase culture to confirm that it remained unchanged (pH between 7.3 and 7.4). Finally, the highest difference of OD_600_ and the difference of the maximum calculated growth rate (v_max_) were calculated by comparing the growth curves of the group exposed to HCN and the control group (no KCN added). Four replicates were used for each experiment. V_max_ was defined as the maximal change of OD_600_ h^−1^ (ΔOD_600_ h^−1^) and was calculated using the Gen5^TM^ Software (Biotek, USA).

### Plant growth experiments

#### Seeds surface sterilization

Plant seeds were surface sterilized using sodium hypochlorite and Tween 20 (Sigma-Aldrich, USA), to remove unwanted microbial contaminants on the seed surface. Different hypochlorite final concentrations (0.5, 1, 2, and 3%) and sterilization times (5, 10, 20, and 30 min) were tested to achieve surface sterility and sufficient (>50%) seed germination efficiency. For maize (*Zea mays* L.) kernels (breed ANJOU 450–FAO 430; Semenarna Ljubljana, Slovenia) 1% NaOCl, 50 μL Tween 20 per 100 mL sterile water and 20 min incubation in a magnetic rotary mixer were determined to be the optimal conditions for material quantity of 100 kernels. For French sorrel (*Rumex scutatus* L.), the optimal conditions were 3% NaOCl 50 μL Tween 20 per 100 mL sterile water and a 30 min mixing incubation for the quantity of 100 seeds. Seeds were washed six times with 100 mL of sterile distilled water and the kernels were aseptically transferred onto a moist sterile filter paper to induce germination. Surface sterilization control was performed after the washing step by transferring the surface sterilized seeds into fresh liquid LB medium (Sigma-Aldrich, USA). Controls were incubated on a rotary mixer at 25°C for 7 days. After the start of seed germination and the breaking of the seed coat, the sterility of the media was monitored daily. This assured that no seed associated bacteria were left present on the surface of or within (potential endophytes) surface-sterilized seeds.

#### Setup of the plant growth experiment

Two types of plants were selected: pioneer plant (French sorrel), with small seed storage capacity and pronounced weathering capabilities dependent on organic acids such as oxalate, and common crop plant (maize; Anjou 450, FAO 430 Semenarna Ljubljana, Slovenia), with large seed storage capacity and adaptation to rich organic soils. Acidic granite sand and basic limestone sand were chosen as the growth substrates. Sterile germinated plant seedlings were aseptically transferred to sterile glass growth tubes containing 8 g of ground bedrock (sand particle size <1 mm). A total of 2 mL of inoculation solution, containing either bacterial cells, nutrients or only distilled water, was added aseptically to the seedlings. Different inoculation solutions were: (*i*) bacterial cells of either of the three tested strains washed with sterile distilled water, (*ii*) an autoclaved mixture of washed cells of all the three strains tested (control I, the negative control for testing the effects of metabolites and nutrients arising from the bacterial cell biomass), and (*iii*) sterile distilled water (control II, the negative control of plant growth without bacteria or external nutrient input). Plants were grown at 25°C under aseptic conditions in a growth chamber with artificial light under a 12 h (day)/12 h (night) light cycle. Young plants were collected after 30 days. Due to the size, morphological characteristics and mass of young plants, maize shoots (leaves and stem) were separated from the roots, while sorrel plants were further analyzed undissected. Plant material was air dried and dry mass was measured. After the plant had been removed from the growth chambers, the test for external contamination and contamination by endophytes was performed by re-isolating the initially inoculated bacterial strains from the growth substrate using KB plate culturing. The identity of the re-isolated bacterial strains was matched with that of the initial inoculating strain based on colony morphology and BOX-PCR profiling (Currie et al., 2007). Contaminated or collapsed plants were removed from further analysis.

### Effect of cyanide on phosphate availability in solution

#### Phosphate colorimetric measurement

The effect of KCN on the concentrations of free phosphate was examined by measuring free phosphate in solution in different scenarios (*see below*). Phosphate concentration was determined using the modified method of the Visocolor ECO kit (Macherey-Nagel, Germany) based on colorimetric detection of products produced in the reaction between ammonium molybdenate and free phosphate (Murphy and Riley, 1962). A SynergyH4 plate reader (Biotek, USA) and clear flat-bottom micro titer plates (Greiner Bio-One, Germany) were used for the measurements. Solutions with known concentrations of KH_2_PO_4_ (Sigma-Aldrich, USA) were used as a quantification standard. A total of 180 mL of sample was firstly thoroughly mixed with 10 mL of reagent “PO_4_-1” (Macherey-Nagel, Germany), secondly with 10 mL of reagent “PO_4_-2” (Macherey-Nagel, Germany) and was then incubated for 10 min at room temperature. The reagent “PO_4_-1” contained 15% sulfuric acid, which adjusted the pH of all samples that we analyzed to 1.6–1.7, as determined by the inoLab pH 730 pH meter (WTW, Germany) and the Sentix^®^ Mic pH electrode (WTW, Germany). Spectrophotometric detection of the colored product was performed at 740 nm. MiliQ water was used as phosphate-free negative control. The linear range of colorimetric measurement was determined to be between 0 and 100 μM of phosphate concentrations, which we implemented in our measurements.

#### Dissolution of granite sand

The dissolution of granite was first examined in KCN solution *in vitro.* The interactions of KCN with the granite mineral sand were monitored indirectly by measuring the conductivity of the incubation solution, which consisted of a series of KCN concentrations (0, 10, 50, 100, 500, or 1000 μM) in miliQ water. A total of 1 g of granite sand was washed three times with 10 mL miliQ water before it was added to 5 mL KCN incubation solution or miliQ water (control 0 μM KCN). The mixture was incubated at 20°C for 7 days on a rotary shaker (250 rpm). The pH of the solution was determined using the inoLab pH 730 pH meter (WTW, Germany) and the Sentix® Mic pH electrode (WTW, Germany). Before further analysis, the solution was centrifuged for 1 min at 10,000 × g. A total of 1 mL of supernatant was collected for the conductivity measurement using DelsaNano HC (Beckman Coulter, USA). The same series of pure KCN solutions was used as negative control. Same amount of liquid sample was collected for phosphate concentration determination by the colorimetric measurement described above.

#### Phosphate availability measurements

The interactions of iron, phosphate and cyanide were examined *in vitro* by: (*i*) sequestering phosphate with iron, using a mixture of KH_2_PO_4_ and FeCl_3_ (both Sigma, USA), (*ii*) sequestering Fe^3+^ ions with KCN (Alfa Aesar, USA), and subsequently adding PO4^3−^ (KH_2_PO_4_) to the mixture and (*iii*) first mixing FeCl_3_ and KH_2_PO_4_ (to prepare iron-phosphate complexes) and subsequently adding KCN to the mixture to sequester iron and indirectly release phosphate from the complexes. Different ratios of FeCl_3_:KH_2_PO_4_ (1:1, 5:1, 10:1, 50:1, 100:1) and KCN:FeCl_3_ (1:1 and 10:1) at different concentrations of FeCl_3_ (10, 100 μM and 1 mM) and KH_2_PO_4_ (10, 100 μM) were examined. The mixture of the first two components was incubated at 20°C for 1 h at 250 rpm on a rotary shaker. Afterwards the third component was added, and the mixture again incubated under the same conditions. The pH of the mixtures was determined using the inoLab pH 730 pH meter (WTW, Germany) and the Sentix® Mic pH electrode (WTW, Germany). For each combination of the final mixture liquid samples were collected to determine the concentration of free phosphate (colorimetric measurement described above).

### Statistical analysis

All statistical analysis was performed using the R software (R Core Team, 2013). Graph plotting was prepared and visualized using the standard R base package (R Core Team, 2013), LibreOffice Calc. (The Document Foundation), and Inkscape (Free Software Foundation).

## Results

### HCN production and its effect on growth of phytopathogenic fungi and bacteria

**Figure 1:**
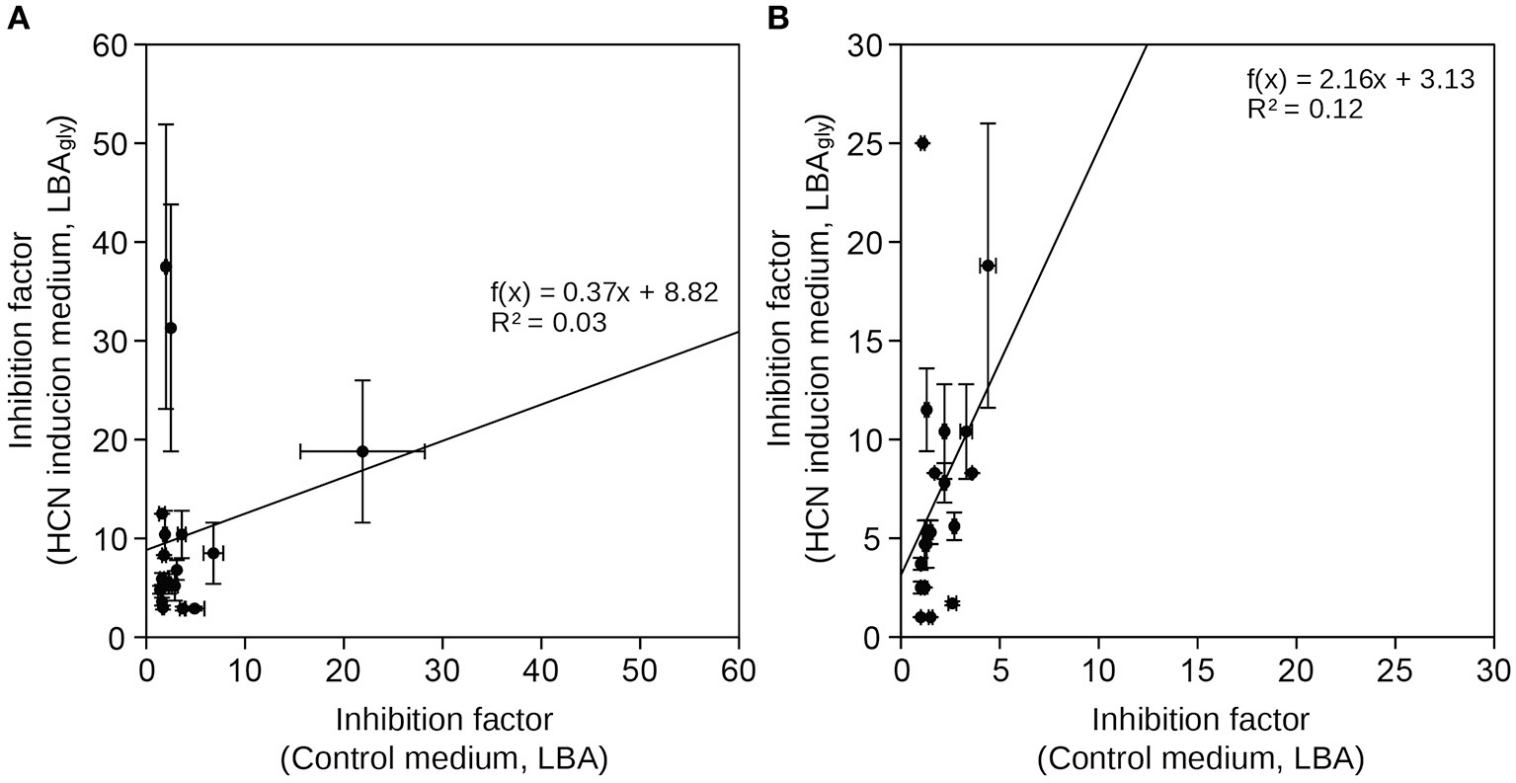
**Comparison of inhibition of growth of phytopathogenic fungi by HCN+ and HCN− rhizobacterial isolates**. Inhibition factors were calculated on control medium, not inducing HCN production (LBA) and on growth medium inducing HCN production (LBA_gly_). **(A)** HCN+ strains **(B)** HCN− strains. Data represents average ± SD (*n* = 4).

**Figure 2:**
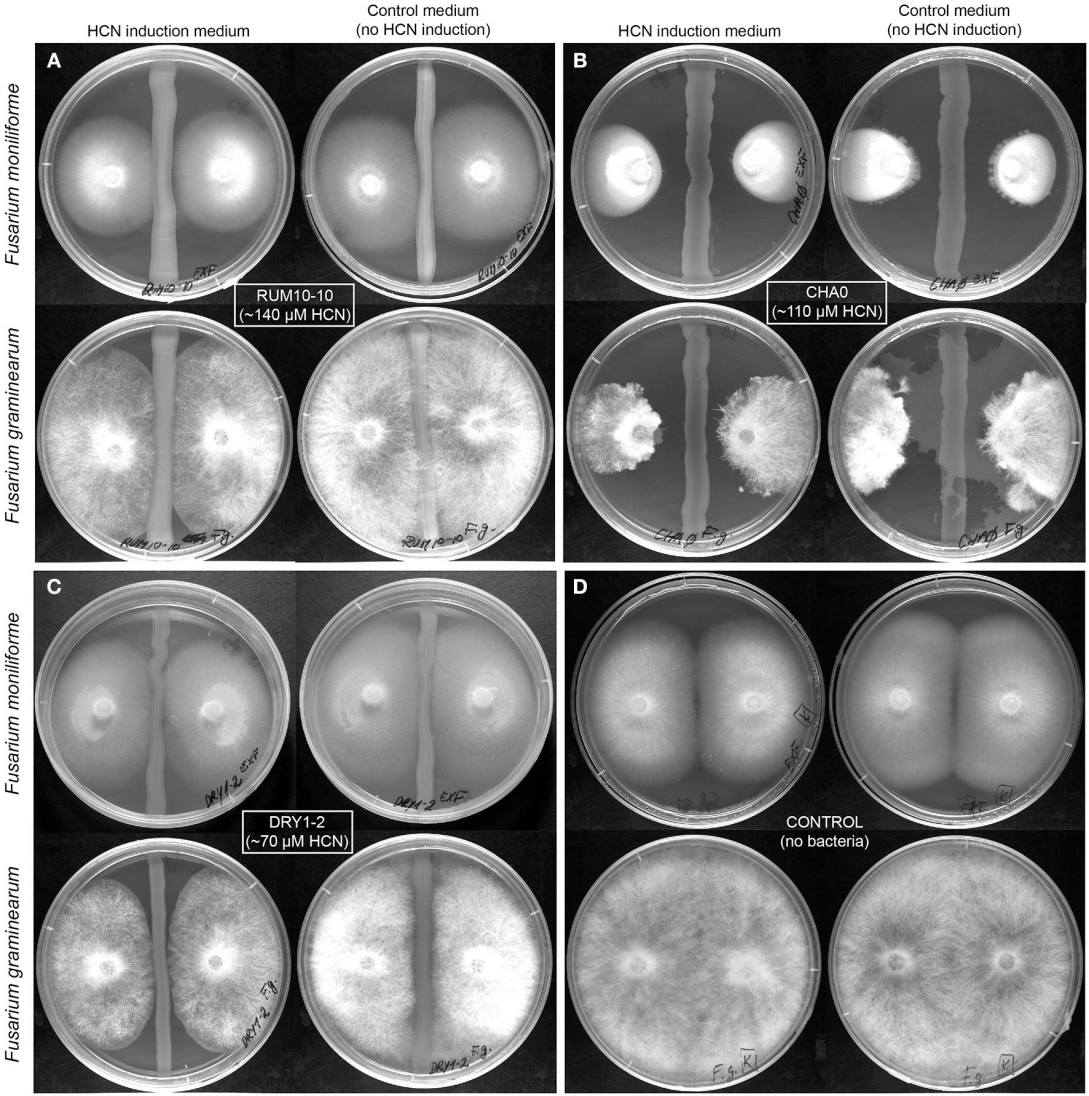
**Inhibition of growth of phytopathogenic *Fusarium* spp. by HP isolates**. Inhibition was tested on HCN induction medium, LBA_gly_ and control medium, and LBA, not additionally inducing HCN production. Bacteria are applied to the middle of the plate in a straight line and fungi are placed on both sides as agar plugs so that the mycelium and the bacteria are not in direct contact. The distance between bacteria and fungal hyphae shows inhibition of fungal growth by bacterial strains. **(A)** HCN+ environmental isolate RUM10-10 (138 μM HCN in liquid culture, l.c.), **(B)** HCN+ strain CHA0 (107 μM HCN in l.c.), **(C)** HCN+ environmental isolate DRY1-2 (HCN production 66 μM in l.c.), **(D)** Control fungal growth without bacteria present.

We selected 10 HCN+ and 9 HCN− environmental isolates and compared their biocontrol potential to the HCN+ strain CHA0, which showed the highest biocontrol potential against both examined phytopathogenic fungi (Table 1, Table S1) and was used as a reference. Two HCN+ strains were weaker HCN producers than strain CHA0 (~ 50–70 μM HCN in pure liquid culture, l.c.), while the remainder were stronger producers (~ 110–190 μM HCN in l.c.; Table 1). We observed no correlation between the intensity with which the HCN+ rhizobacteria could produce HCN *in vitro* and the inhibition of growth of phytopathogenic fungi or bacteria (*R* = 0–0.15; Figure 1). HCN+ strains were generally not strong inhibitors of growth and no increase of biocontrol by HCN+ strains was observed on LBA_gly_, which additionally induced HCN production (Figures 1, 2). In contrast, many HCN− strains showed antifungal activity, which was increased on LBA_gly_ medium (Table 1, Figure 1). The calculated inhibition factors of HCN+ strains did not differ from those of HCN− strains (*P* = 0.21 and 0.13 for *F. graminearum* and *F. moniliforme*, respectively). Several HCN+ strains, while inhibiting the growth of fungi, failed to inhibit the growth of phytopathogenic bacteria. Only three out of 11 HCN+ strains inhibited the growth of *P. syringae* pv. *syringae*, and only one, strain DRY1-2 (not among the strongest HCN producers), inhibited the growth of two other bacterial pathogens, *P. syringae* pv. *coronafaciens*, and *E. carotovora* pv. *carotovora* (Table 1). Inhibition of phytopathogenic bacteria was observed only on the control LBA medium, which did not stimulate HCN production.

**Table 1:** **Inhibition of growth of plant pathogenic fungi and bacteria by environmental isolates**.

	**Strain**	**HCN production^a^ (μM)**	**Pyoverdine production**	**Catechol production**	**Hydroxamate production**	**Carboxylate production**	**Inorganic PO_4_ solubilization^b^**	**General IF—Fungi^c^*F. graminearum/F. moniliforme***	**HCN mediated IF—Fungi^d^*F. graminearum*/*F. moniliforme***	**General inhibition^e^ Pathogenic bacteria**
HCN+ rhizobacterial isolates	RUM5-1	55	+	+	−	+	+	2.70|1.32	2.06|4.00	0/4
	DRY1-2	66	−	+	−	−	+	2.50|1.72	13.33|1.71	3/4
	CHA0	107	+	+	+	+	−	25.00|25.00	ND^d^|ND^d^	1/4
	RUM2-2	118	+	+	+	+	+	3.57|1.79	2.80|2.95	0/4
	DRY9-8	132	+	+	+	+	−	2.86|4.76	1.67|0.60	0/4
	RUM10-10	138	−	−	+	+	−	20.00|1.89	0.83|5.30	1/4
	K9-7	138	+	+	+	+	+	3.13|1.59	2.13|3.71	0/4
	R10-1	153	−	+	+	+	+	1.96|2.27	25.50|2.32	0/4
	R7-1	156	+	+	−	+	+	1.52|1.64	8.25|2.18	0/4
	R6-5	175	−	−	+	+	−	6.67|1.82	1.15|4.58	0/4
	R6-8	185	+	+	−	+	+	3.70|1.43	0.77|3.33	0/4
HCN− rhizobacterial isolates	DRY1-10	0	+	+	−	+	+	1.15|2.17	3.95|4.60	0/4
	DRY4-5	0	+	+	−	+	+	1.30|1.33	3.50|8.33	0/4
	RUM3-2	0	−	+	−	+	+	1.52|1.67	3.47|5.00	0/4
	RUM3-9	0	+	+	−	+	+	1.00|1.09	2.50|23.00	0/4
	K5-9	0	+	+	−	+	+	3.33|4.35	3.00|3.83	0/4
	K7-8	0	−	+	−	−	+	1.00|1.23	1.00|2.03	0/4
	K7-10	0	−	+	−	−	+	1.49|1.00	0.67|3.70	0/4
	R2-1	0	−	+	−	+	−	3.57|2.22	2.33|3.46	0/4
	R2-9	0	−	+	−	+	−	2.63|1.54	0.66|3.42	0/4

a Concentration of biogenic HCN produced by environmental strains in overnight pure liquid culture.

b Indication of organic acid production.

c General Inhibition Factor assessed on control medium (LBA), not inducing HCN production. IF calculated by dividing fungal colony diameter in the presence of bacterial environmental isolates with diameter of fungal colony in control set up without bacteria.

d HCN mediated Inhibition factor assessed on culture medium supplemented with glycine to induce HCN production. IF calculated by dividing fungal colony diameter in the presence of bacterial environmental isolates with diameter of fungal colony in control set up without bacteria.

e Number of phytopathogenic bacteria inhibited by environmental isolate (out of 4 tested) on LBA, where additional HCN production was not induced. On LBA_gly_, which additionally stimulates HCN production, no inhibition of phytopathogenic bacteria was observed (HCN mediated inhibition was 0/4 for all rhizobacterial strains tested). IF, Inhibition factor; HCN+, environmental isolate able to produce HCN; HCN−, environmental isolate not able to produce HCN. Gray area labels HCN+ strain CHA0, which was used as standard for comparison with other HCN+ strains.

### Effect of HCN on growth of rhizospheric environmental isolates

**Figure 3:**
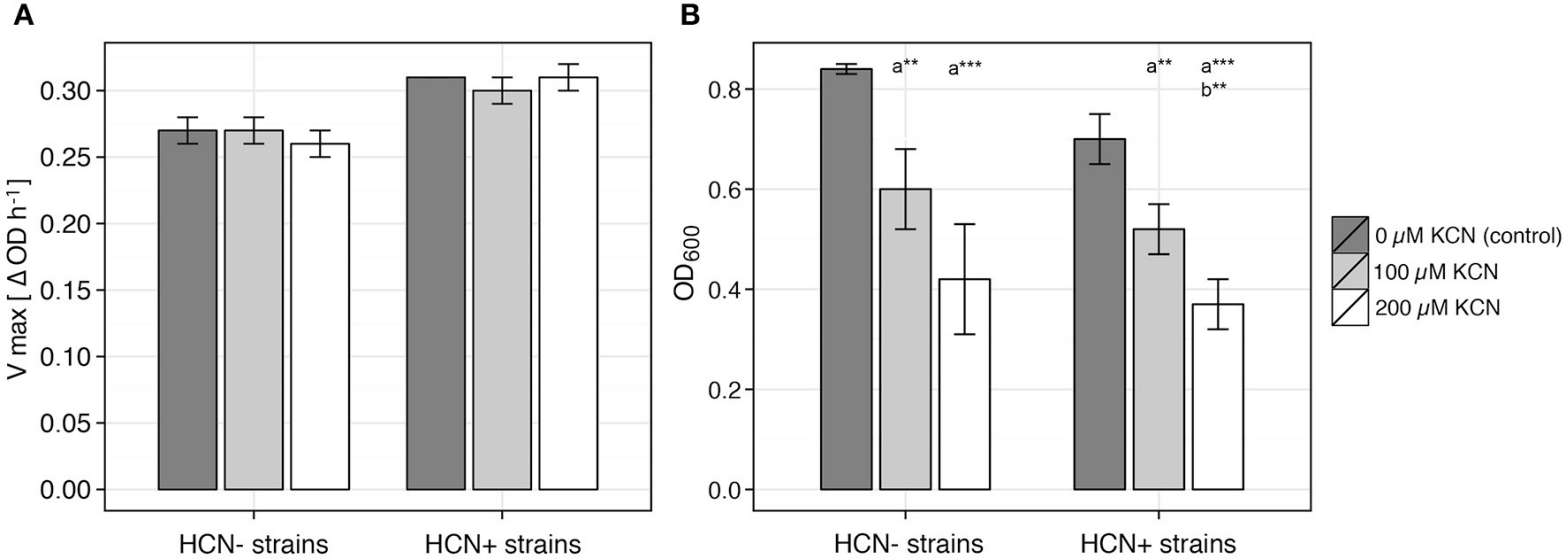
**Effect of cyanide on growth of plant-associated bacterial isolates**. Growth characteristics of 4 HCN+ and 4 HCN− environmental isolates were examined in growth medium supplemented with KCN and in control medium without KCN, to determine the deviation of growth affected by cyanide from control growth. V_max_ (maximum calculated growth rate, ΔOD h^−1^) and OD_600_ were used to characterize culture growth. **(A)** Average V_max_. **(B)** Average OD_600_ measured at maximum deviation from control group. Data represented as average ± SE of 4 strains in each group and 4 replicate growth experiments for each strain. Student *t*-test, two-tailed; ^***^P < 0.01, ^**^P < 0.05, was used for comparison of: (a) growth influenced by KCN supplement (100 or 200 μM KCN) with growth of control group (0 μM KCN) and (b) growths influenced by different concentrations of KCN supplement within one strain phenotype (HCN+ or HCN−).

Using 4 HCN+ and 4 HCN− environmental isolates, we determined the MIC of HCN to be between 1 and 2 mM for all the strains tested. When KCN was added to the medium at concentrations that were also produced by the strongest HPB in *in vitro* liquid culture (100–200 μM), all the strains continued to grow without a significantly reduced maximal calculated growth rate (V_max_; ΔOD_600_h^−1^; *P* > 0.36, Student *t*-test, two-tailed; Figure 3). We observed that the addition of cyanide caused a temporal shift of the growth curves, as demonstrated by the reduced OD_600_ (Figure 3, Figure S3) in the early growth stages of the liquid culture, before V_max_ was reached (Figure S4). The maximum difference of OD_600_ was temporally correlated with v_max_ and always occurred up to 1.2 h (based on our sampling interval) before it (*R* = 0.963, *P* < 0.001; Figure S5). We found that the growth of one HCN− strain (RUM3-2), out of the four tested, was even less affected by the presence of cyanide than all the HCN+ strains (Figure S4).

### Plant growth promotion by isolated HCN-producing bacteria

**Figure 4:**
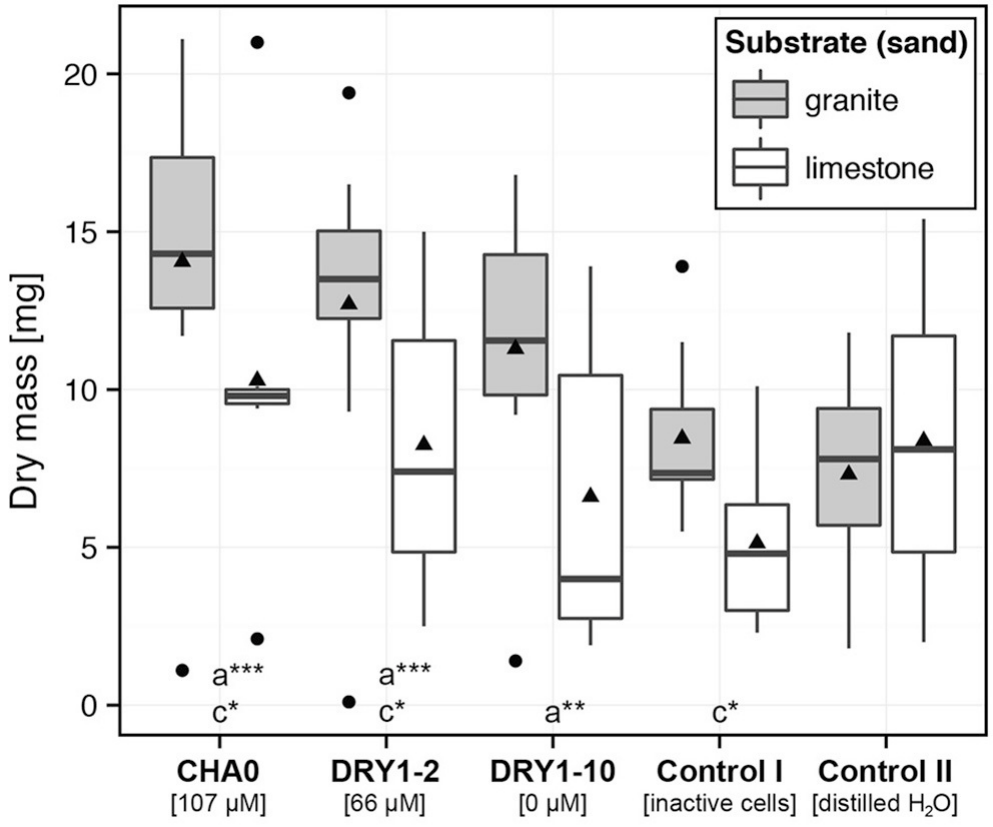
**Growth of pioneer plant French sorrel (*Rumex scutatus* L.) on substrates inoculated with bacterial isolates**. Dry mass of young plants, growing aseptically on sterile granite (*gray*) or limestone (*white*) sand. The growth substrate was inoculated with either the cell suspension of a single bacterial strain, the autoclaved mixture of cells of all three strains (CHA0, DRY1-2, DRY1-10) (control I), or sterile distilled water (control II). HCN+ strain CHA0, 107 μM HCN in liquid culture (l.c.); HCN+ strain DRY1-2, 66 μM HCN in l.c.; HCN^−^ strain DRY1-10, 0 μM HCN in l.c. Data are represented as a box plot, (*n* = 10) with 1st and 3rd quartiles, median (horizontal line) and average (black triangle). Whiskers represent the max. and min. at the 1.5 times above the interquartile range of the data and points show outliers above both maxima. Mann-Whitney U-test, two-tailed; ^***^P < 0.01, ^**^P < 0.05, ^*^P < 0.1, comparison was made between plant growth on bacteria-inoculated substrate and plant growth in control II on (a) granite and (b) limestone and (c) between different types of substrate of the same treatment.

**Figure 5:**
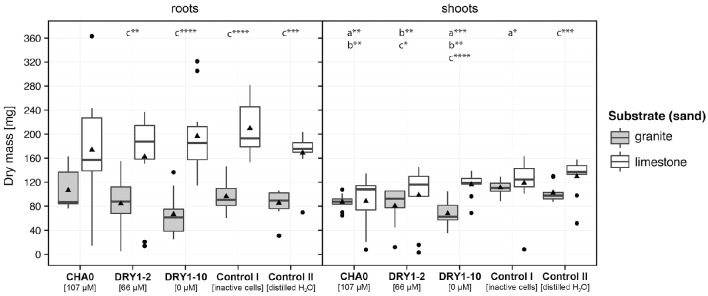
**Growth of crop plant maize (*Zea mays* L.) on substrates inoculated with bacterial isolates**. Dry mass of roots and shoots of young plants, growing aseptically on sterile granite (*gray*) or limestone (*white*) sand. The growth substrate was inoculated with either the cell suspension of a single bacterial strain, the autoclaved mixture of cells of all three strains (CHA0, DRY1-2, DRY1-10; control I), or sterile distilled water (control II). HCN+ strain CHA0, 107 μM HCN in liquid culture (l.c.); HCN+ strain DRY1-2, 66 μM HCN in l.c.; HCN^−^ strain DRY1-10, 0 μM HCN in l.c. Data are represented as a box plot, (*n* = 10) with 1st and 3rd quartiles, median (horizontal line) and average (black triangle). Whiskers represent the max. and min. at the 1.5 times above the interquartile range of the data and points show outliers above both maxima. Mann-Whitney U-test, two-tailed; ^****^P < 0.001, ^***^P < 0.01, ^**^P < 0.05, ^*^P < 0.1; comparison was performed between plant growth on bacteria-inoculated substrate and plant growth in control II on (a) granite, (b) limestone and (c) between different types of substrate of the same treatment.

We observed a significant increase of dry mass (DM) of young French sorrel plants, but only when they were growing on granite and were treated with viable bacteria. The most prominent effect, compared to the controls, was attributed to the HCN+ strain CHA0 (~ two-fold increase of DM compared to both control treatments), followed by the HCN+ strain DRY1-2 (~1.7-fold increase of DM compared to both control treatments) and finally the HCN− strain DRY1-10 (Figure 4). Additionally, only HCN+ strains (CHA0 and DRY1-2), significantly increased the DM of plants when granite substrate was compared to limestone (*P* < 0.1; Mann-Whitney U-test, two-tailed, Figure 4). On the contrary, we observed no effect of HCN+ strains and viable bacteria in general, on the growth of root or shoots of maize plants (Figure 5). Generally, maize growth was poorer on granite than on limestone, but the lowest DM of maize plants was observed in treatments involving HCN− strain DRY1-10 (*P* < 0.001; Mann-Whitney U-test, two-tailed, Figure 5).

Analysis of seed and plantlet dry mass showed that dry mass of maize kernel was almost 200-times larger than the seed of French sorrel. The DM of 30 day old maize plantlets was comparable to that of kernels (mass increase factor = 0.9 ± 0.3), while the DM of French sorrel plantlets increased on average more than five times (factor of 5.5 ± 0.5) relative to the dry mass of seeds (Table S2).

### Effect of cyanide on mineral dissolution and phosphate availability

**Figure 6:**
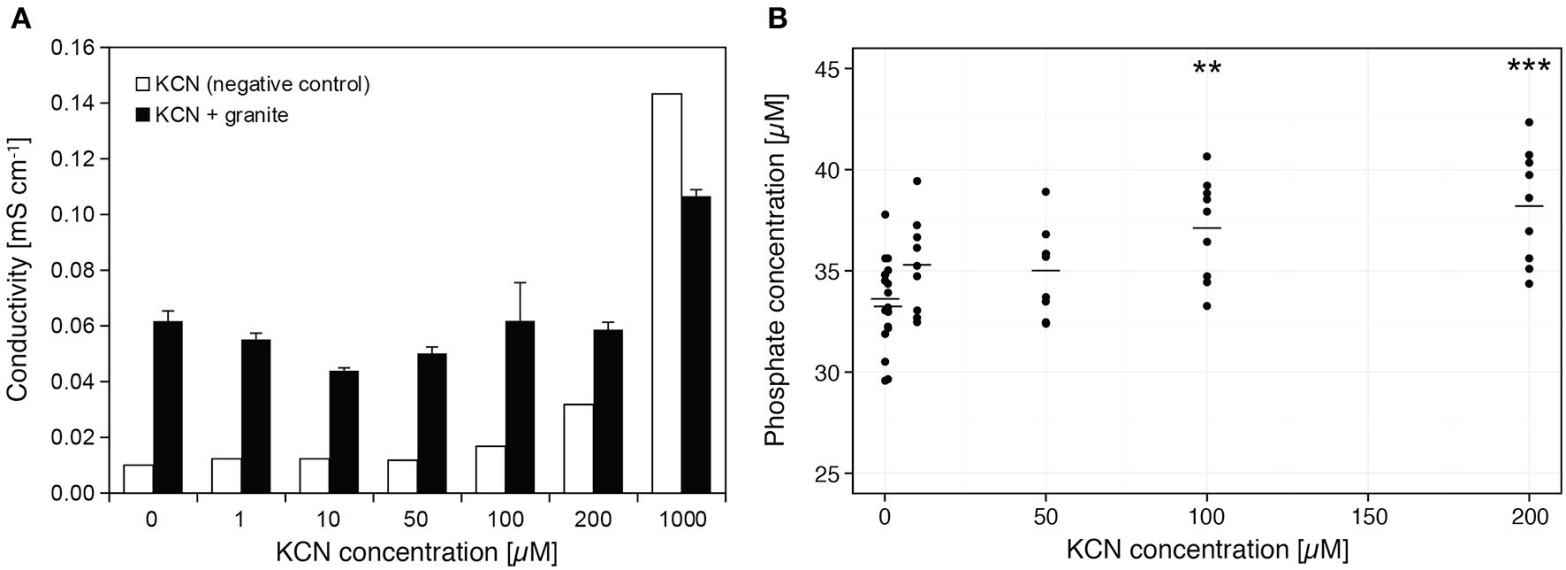
**Dissolution of granite in KCN and release of ${\text{PO}}_{4}^{3-}$**. **(A)** Dissolution of granite at each KCN concentration was assessed by measuring conductivity of solution that was incubated with the granite sand. Pure KCN solution was used as a negative control. **(B)** Released phosphate was determined in solutions containing granite sand at each KCN concentration. Data represent raw measurements (*n* = 9), line represents average; Mann-Whitney U-test, two-tailed ^***^P < 0.01, ^**^P < 0.05 was used for comparison of each dataset with control (0 μM KCN). The pH values at the beginning of the experiment ranged from 6.9 (0 μM) to 7.8 (200 μM) and at the end of the experiment they were in the range 7.6–8.9 (pH level does not correspond to KCN concentration).

When cyanide reacts with granite under *in vitro* conditions, a gradual increase of conductivity with increased KCN concentration is observed. This increase was notably lower when compared to negative control of pure KCN solution (Figure 6A). Phosphate release from granite material was also confirmed, its quantity depending on the increasing cyanide concentration (Figure 6B). Concentrations of free phosphate were significantly larger in solutions containing 100 or 200 μM KCN compared to negative control containing no KCN (*P* < 0.05 and *P* < 0.01, respectively; Mann-Whitney U-test, two-tailed). The pH of the solutions at the beginning of the experiment ranged from 6.9 (0 μM) to 7.8 (200 μM) and at the end of the experiment they were determined to be in the range 7.6–8.9, but not corresponding to the concentration of KCN.

**Figure 7:**
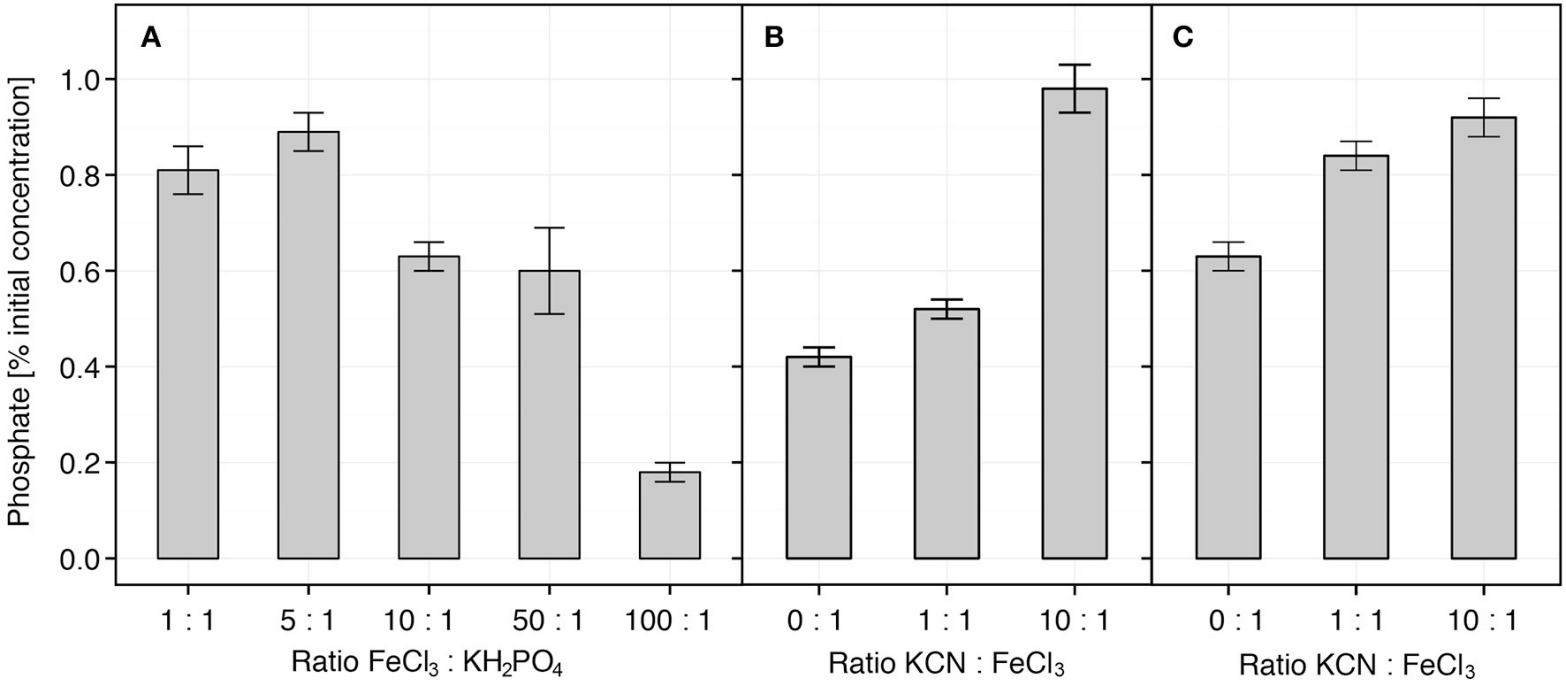
**Availability of PO_4_*in vitro*.** Availability of PO_4_ in solution was examined *in vitro* in the presence of iron and cyanide in different combinations. Percentage of initial ${\text{PO}}_{4}^{3-}$ concentration was determined after FeCl_3_ and cyanide (KCN) had been added. **(A)** ${\text{PO}}_{4}^{3-}$ concentration in solution is reduced after addition of iron. The initial concentration of KH_2_PO_4_ was 10 μM. **(B)** When cyanide (KCN) is added to FeCl_3_ before KH_2_PO_4_, the concentration of ${\text{PO}}_{4}^{3-}$ in solution is not reduced. KH_2_PO_4_ initial concentration was 10 μM; FeCl_3_:KH_2_PO_4_ ratio was 10:1. **(C)** KCN increased release of ${\text{PO}}_{4}^{3-}$ from Fe-PO_4_ complexes after it was added to pre-prepared mixture of FeCl_3_ and KH_2_PO_4_. Initial KH_2_PO_4_ concentrations was 10 μM; the FeCl_3_:KH_2_PO_4_ ratio was 10:1. Each mixture was incubated for 60 min. Data represents average ± SE (*n* = 3–9). The pHs of FeCl_3_-KH_2_PO_4_ mixtures ranged from 3.1 (FeCl_3_:KH_2_PO_4_ = 100:1) to 4.7 (1:1). Mixtures of Fe-PO_4_-KCN ranged from 3.9 (FeCl_3_:KCN = 1:1) to 9.4 (1:10).

Further *in vitro* analysis involving pure chemical compounds showed that the concentration of dissolved phosphate (KH_2_PO_4_) was partially reduced in the presence of FeCl_3_ at Fe:PO_4_ ratios of 1:1, but markedly (up to 4-times) at ratio 100:1 (Figure 7A). The pHs of the mixtures ranged from 4.7 (1:1) to 3.1 (100:1). The effects of KCN could generally not be observed when equal quantities of iron and phosphate (Fe:PO_4_ = 1:1) were used (Figure S6), and thus a higher Fe:PO_4_ ratio (10:1) was applied. When FeCl_3_ was first mixed with different amounts of KCN and the mixture was subsequently mixed with KH_2_PO_4_ (Fe:PO_4_ = 10:1), we observed that only at CN:Fe ratio of 10:1, the concentration of free phosphate in solution was not reduced (Figure 7B). On the contrary, when KCN was added to the FeCl_3_-KH_2_PO_4_ mixture (Fe:PO_4_ = 10:1), the concentration of phosphate was already restored at CN:Fe ratio of 1:1 and even more so at ratio 10:1 (1.1–1.3 times, *p* = 0.07) (Figure 7C). The pH of KCN-FeCl_3_ mixture was determined to be 3.9 (CN:Fe = 1:1) and 9.4 (at 10:1). The colorimetric reaction did not show false positive signals in the absence of phosphate when KCN and FeCl_3_ were mixed at the highest, 10:1, ratio (1 mM KCN, 100 μM FeCl_3_; Figure S6B).

## Discussion

Our plant growth experiments show that on the granite-based sand substrate the inoculation with HCN+ strains improves the growth of French sorrel plants. The observed improved growth of French sorrel in our experiments (Figure 4), cannot be caused by the biocontrol activity of the added bacterial strains or the development of a systemic resistance, since the plants were growing aseptically. In addition, the potential for HCN production of our rhizobacterial strains (maximal production in optimal *in vitro* conditions) failed to correlate with their biocontrol activity against pathogenic fungi or bacteria (Figure 1). We thus focused on our alternative hypothesis, in which HCN acts as a weathering and metal-complexing agent in the substrate, increasing nutrient availability. In the soil, HCN can complex either the excess of micro-elements (e.g., Fe, Cr, Mo, Co) or sequester them from compounds like phosphates, with which these micro-elements usually form insoluble precipitates (reviewed in Dunbar and Heintz, 1997). In our *in vitro* experiments on ground granite sand, we showed that cyanide indeed forms insoluble complexes with the granite mineral substrate, as demonstrated by the decrease of KCN solution conductivity in the presence of granite sand (Figure 6A). Moreover, increased availability of phosphate in solution with increasing cyanide concentration (Figure 6B) was observed, demonstrating that under *in vitro* conditions, available phosphate could have an important role in promoting the growth of French sorrel plants. Depending upon soil type, phosphate availability is among one of the most important limiting factors affecting plant growth (Sanyal and De Datta, 1991). In granite sand, the availability of phosphate is influenced by its precipitation by metals such as iron or aluminum (Chabot et al., 1996; Taunton et al., 2000), while in calcareous soils, phosphate precipitation can be influenced by high levels of calcium (Chabot et al., 1996). Since HCN needs iron to form complexes with calcium, and since the Ca:PO_4_ ratio is extremely high in a limestone based environment, we could not detect any positive growth effect of cyanogenic rhizobacteria on limestone sand (Figures 4, 5). On the other hand, though HCN does not precipitate aluminum (Bergstrom, 1924), it can effectively form complexes with iron, as demonstrated in alpine silicate soils (Frey et al., 2010; Wongfun et al., 2014). Here, a physiological response is implied by the fact that HCN production is induced by iron (Bakker and Schippers, 1987; Keel et al., 1989; Voisard et al., 1989) and that it is under strong influence of quorum sensing (Pessi and Haas, 2000). The latter is likely to happen particularly in the rhizosphere, where root exudation promotes high bacterial counts.

With our *in vitro* experimental system using KCN, FeCl_3_, and KH_2_PO_4_ we have demonstrated indirectly that cyanide interacts with iron, probably forming Fe–CN complexes, as previously described by Wongfun et al. (2014). Unlike HCN, which is a weak acid and the product of the HCN-synthesis pathway (Blumer and Haas, 2000), KCN is a salt, which upon dissociation increases the alkalinity of the solution and thus the solubility of metals. Judging by our results, the pH is not the predominant factor, as it is not consistently associated with KCN concentration (Figure 6) and because cyanide interacts with iron at low and high pH (Figure 7). Although no data were previously available showing that HCN either prevents the complexation of ${\text{PO}}_{4}^{3-}$ by Fe^3+^ or that ${\text{PO}}_{4}^{3-}$ is released from Fe-PO_4_ complexes, we concluded from our results that the interaction of iron and cyanide can increase the availability of phosphate. According to our results, the second scenario, in which KCN causes the release of ${\text{PO}}_{4}^{3-}$ from Fe-PO_4_ complexes was more favorable, since it is needed less KCN for every released phosphate ion (Figure 7C). This observation has important biological implications, assuming the level of HCN production in the rhizosphere is much lower compared to ideal *in vitro* conditions, where the HCN precursor glycine, is in excess. It should be noted that the conditions for HCN production might even be highly favorable in the rhizosphere, since with an efflux at 20–65 μmol g^−1^ root DW h^−1^, glycine has been recorded to be one of the predominant amino acids of root exudates (Lesuffleur et al., 2007). At the same time, in the rhizosphere, the amount of soluble iron is increased and exceeds that of phosphate, because plants and bacteria excrete organic acids and siderophores (reviewed in Kiczka et al., 2010), which results in Fe-PO_4_ complexation. Without a mechanism for removal of excess iron, like irreversible complexation by HCN, the production of different siderophores and organic acids (Table 1) cannot regulate the interaction of excess iron with phosphate. Consequently, we argue that the production of siderophores or the ability of the rhizobacteria to mobilize inorganic phosphate alone (Table 1) cannot explain the observed differences in plant growth (Figure 4).

The chemical characteristics of the substrate call for high local concentrations of HCN to be ensured. In granite based mineral soils with pH = ~5 (far below the pKa of HCN—9.2; Grant, 1969) the volatile HCN molecule and not the CN^−^ ion is the favored form, indicating either the release of HCN from the system or immediate complexation with components of the soil. We thus expect that HCN complexation of iron will have a biologically relevant effect only in local niches close to the roots and mostly after Fe-PO_4_ complexes are formed, since bacteria probably produce HCN in lower concentrations than necessary, at least 75 μmol g^−1^ soil for neutral pH and up to 3-times more at pH = 6, to complex all the iron in the bulk mineral soil within the rhizosphere (450 μmol g^−1^ for granite-based mineral soil; Frey et al., 2010). If phosphate is made available in the vicinity of both organisms, then both bacteria and plants could benefit from this complexation process.

Contrary to the results of experiments involving the French sorrel, we could not see any positive effects of rhizobacteria on the growth of maize plants on both mineral substrates tested (Figure 5). We hypothesize that young plants like French sorrel, with little nutrient supply in the seed, depend on nutrient availability in the substrate soon after germination and in this respect, French sorrel has much smaller seed size than maize (Table S2). We thus expect the French sorrel seedlings to be more affected by substrate treatments (i.e., inoculation of strains, input of nutrients, watering) compared to maize seedlings (Figures 4, 5). French sorrel is also adapted to mineral soils and is involved in the weathering process by excreting many products and exudates such as organic acids (predominantly oxalic acid) and chelating agents (Hinsinger, 2001; Göransson et al., 2014). Maize, on the other hand, is in its early development provided with nutrients from the large starchy storage tissue, the endosperm (Kiesselbach, 1999). This is demonstrated by the kernel-to-plant transfer of the biomass (Table S2; Cooper and MacDonald, 1970). As it does not depend on external resources in this early part of development as much as the French sorrel, the processes occurring in the substrate are not reflected in the biomass. Secondly, maize agricultural hybrid cultivars, are adapted to agricultural rich organic soils, where phosphate is made available by mineralization of organic materials and not by weathering of minerals. These cultivars are also not specialized for growth on mineral sand as are the alpine pioneer plants, though organic acid exudation from roots, in lower concentrations and different composition has been well-documented in maize (summarized from (Kiczka et al., 2010) and Ström et al., 2002).

HCN interrupts the activity of metallo-enzymes (Cooper and Brown, 2008) and reacts with keto compounds and Schiff-base intermediates (reviewed in Solomonson, 1981), and as a result, it can inhibit the functioning of many enzymes or protein carriers and in specific cases probably can inhibits the growth of certain organisms. However, based on the results of this study, and work presented by others (Alström and Burns, 1989; Pal et al., 2000; Kremer and Souissi, 2001; Rudrappa et al., 2008; Blom et al., 2011), we conclude that HCN is hardly a universal biocontrol agent. If HCN indeed presents an advantage over competitor microorganisms in the rhizosphere and thus indirectly promotes the growth of host plants, it would need to specifically inhibit only the growth of phytopathogens. Firstly, we have clearly shown that there is no correlation between the amount of HCN produced by a particular strain and its ability to inhibit the growth of phytopathogenic bacteria or fungi (Figures 1, 2, Table 1). Secondly, HCN does not inhibit the growth of HCN− rhizobacteria *in vitro* (Figure 3). Since the MIC of cyanide was determined to be 10 times higher than concentrations otherwise produced by HPB in ideal *in vitro* conditions and since some HCN− rhizobacteria (e.g., strain RUM3-2) were completely unaffected by HCN, we conclude: (*i*) MIC concentrations can hardly be reached in natural systems and (*ii*) HCN is not toxic for plant-associated rhizobacteria (Figure 3, Figures S3, S4). Indeed, the possibilities for metabolic assimilation of HCN have been described in bacteria (Knowles, 1976), and confirm that HCN does not represent a toxicity factor for them. Previous studies do not address this issue and a clear connection between HCN production and biocontrol of phytopathogens was never unequivocally established. Several earlier studies, focusing on PGP, merely acknowledge the presence of HCN+ strains among rhizospheric isolates (e.g., Chabot et al., 1996; Marques et al., 2010), but the mechanism of HCN action is not described. Keel et al. (1997) suggested that in the rhizosphere, HCN could bind iron, though this idea was focused on the competition for available iron with phytopathogens and was thus also associated to exert biocontrol. In the rhizosphere, HCN is unlikely to be used for competition, since to bind iron, it would need to compete with the siderophores produced by the HCN producer's competing microorganisms. Both HCN and siderophores have similar Fe-complex stability constants (~10^43^), but the latter are produced at much higher rate and have generally higher specificity for Fe^3+^ than HCN does (summarized from Dzombak et al., 2006 and Domagal-Goldman et al., 2009). Finally, what our *in vitro* experiments cannot exclude, is the possibility that HCN induces systemic resistance in some plants (Wei et al., 1991), making them resistant to phytopathogen attack. In regards to HCN's toxicity levels and plant functioning, it is evolutionary and ecologically hard to describe the mechanisms, in which HCN produced by bacteria would act as an active mediator of metabolic processes in plants. Much work on the proposed regulatory functions of HCN in plants (Siegień and Bogatek, 2006) will be needed in the future to address this issue.

**Figure 8:**
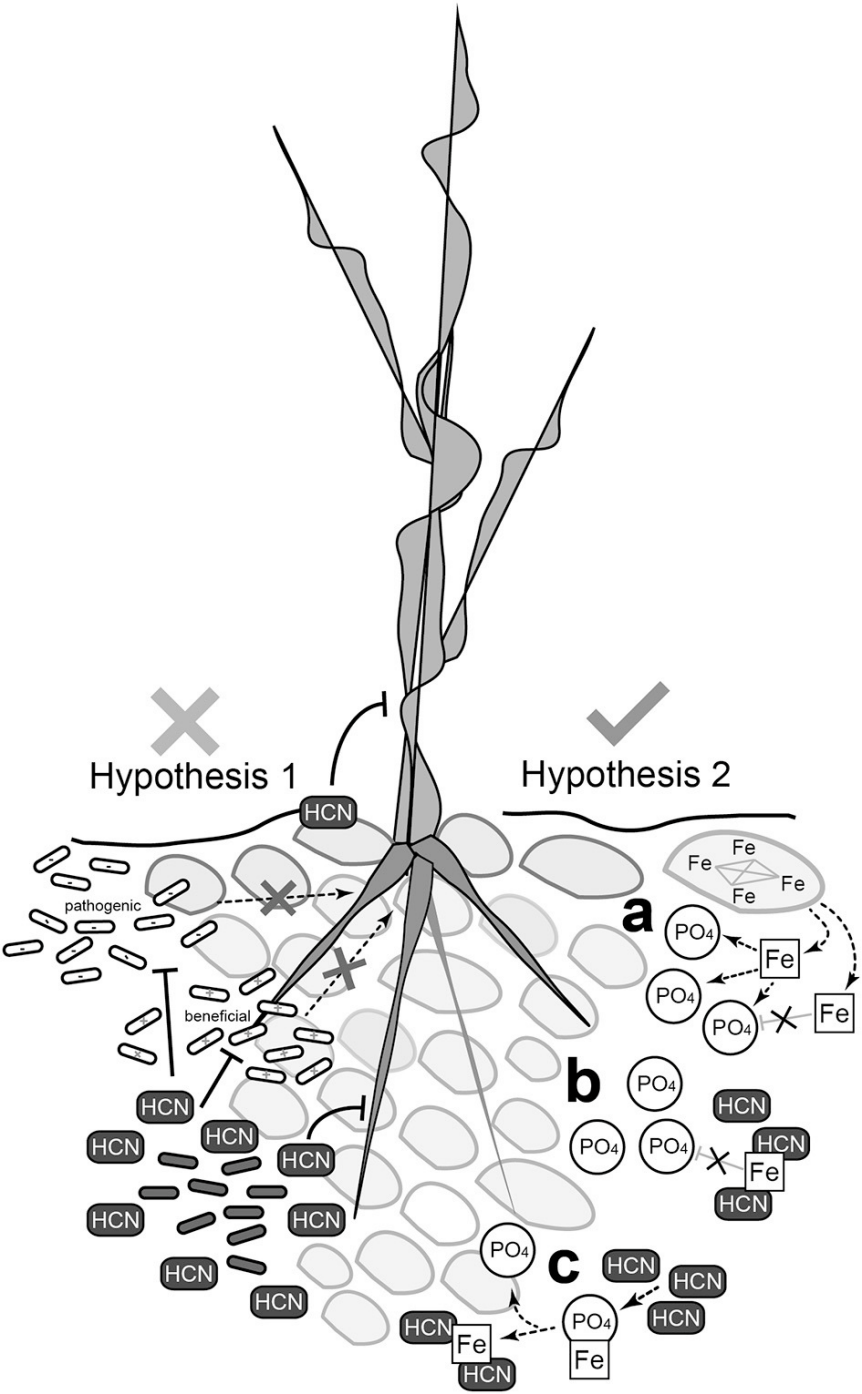
**Schematic representation of the two hypotheses tested and summary of possible mechanisms of HCN's activity**. The biocontrol hypothesis for real environmental systems is questionable, since HCN's toxicity, specifically targeting phytopathogens, is difficult to support (*left*). A new concept, in which HCN is involved in geochemical processes and regulation of nutrient availability, is much more suitable to explain the role of HCN production by rhizobacteria. **(A)** Iron (Fe) binds free phosphate (PO_4_) making it insoluble and thus unavailable for bacteria and plants. **(B)** Cyanide (HCN) sequesters iron, preventing it from binding free phosphate. Phosphate remains available in solution for bacteria and plants. **(C)** Phosphate is released from the iron-phosphate complex after cyanide binds iron forming the Fe-CN complex.

In conclusion, we propose a new role for HCN production by rhizospheric bacteria. HCN increases phosphate availability for rhizobacteria and plant hosts, and this is especially applicable to oligotrophic alpine environments (Figure 8). Increased availability of nutrients by which HPB promote the growth of pioneer plants is possibly the mechanism. With increased frequency of nutrient deficient soils and exposed areas produced by deglaciation (Kaser et al., 2004; Gurung and Bajracharya, 2012) and other types of erosion (Stanchi et al., 2013), a better understanding of the involvement of HPB in geochemical processes will be of great importance in the future.

## Author contributions

Both authors have contributed to design of the work, acquisition, analysis, and interpretation of data for the work and preparation of the manuscript.

### Conflict of interest statement

The authors declare that the research was conducted in the absence of any commercial or financial relationships that could be construed as a potential conflict of interest.
