# Supplementary material for: Hydrogen Cyanide in the Rhizosphere: Not Suppressing Plant Pathogens, but Rather Regulating Availability of Phosphate
Source: Front Microbiol. 2016 Nov 18;7:1785. doi: 10.3389/fmicb.2016.01785 (PMC5114478; doi:10.3389/fmicb.2016.01785)
Supplement: Supplementary file 1 [file Table1.pdf]

Hydrogen cyanide in the rhizosphere: not suppressing plant pathogens, but rather regulating availability of phosphate

Tomaž Rijavec[1,2] and Aleš Lapanje[1,2,3,*]

[1] Institute of Metagenomics and Microbial Technologies, Clevelandska ulica 19, 1000 Ljubljana, Slovenia

[2] Department of Environmental Sciences, Jožef Stefan Institute, Jamova cesta 39, 1000 Ljubljana, Slovenia

[3] Remote Controlled Theranostic Systems Lab, Saratov state university, Astrakhanskaya 83, Saratov, Russian Federation

Correspondence: Aleš Lapanje, Institute of Metagenomics and Microbial Technologies, Clevelandska ulica 19, 1000 Ljubljana, Slovenia, 00 386 68 604 979

SUPPLEMENTARY TABLES

S1-S2

TABLE S1 | Biocontrol potential of HCN+ and HCN- environmental strains against two fungal phytopathogens from the genus *Fusarium*.

				Fusarium graminearum		*Fusarium moniliforme*	
	Strain	HCN production[1]	Test Medium[2]	Diameter (cm)	Inhibition[3]	Diameter (cm)	Inhibition[3]
HCN+ strains	**RUM5-1**	55 µM	no induction	9.25 ± 0.50	2.06	19.00 ± 1.15	4.00
			**HCN induced**	4.50 ± 0.58		4.75 ± 0.50	
	**DRY1-2**	66 µM	no induction	10.00 ± 0.00	13.33	14.50 ± 0.58	1.71
			**HCN induced**	0.75 ± 0.50		8.50 ± 0.58	
	**CHA0**	107 µM	no induction	0.00 ± 0.00	Not determined	0.00 ± 0.00	Not determined
			**HCN induced**	0.00 ± 0.00		0.00 ± 0.00	
	**RUM2-2**	118 µM	no induction	7.00 ± 0.82	2.80	14.00 ± 0.82	2.95
			**HCN induced**	2.50 ± 0.58		4.75 ± 0.50	
	**DRY9-8**	132 µM	no induction	8.75 ± 0.96	1.67	5.25 ± 0.96	0.60
			**HCN induced**	5.25 ± 1.89		8.75 ± 0.50	
	**RUM10-10**	138 µM	no induction	1.25 ± 0.50	0.83	13.25 ± 1.50	5.30
			**HCN induced**	1.50 ± 0.58		2.50 ± 0.58	
	**K9-7**	138 µM	no induction	8.00 ± 0.00	2.13	15.75 ± 1.50	3.71
			**HCN induced**	3.75 ± 0.50		4.25 ± 0.50	
	**R10-1**	153 µM	no induction	12.75 ± 0.50	25.50	11.00 ± 0.82	2.32
			**HCN induced**	0.50 ± 0.58		4.75 ± 0.96	
	**R7-1**	156 µM	no induction	16.5 ± 3.00	8.25	15.25 ± 0.96	2.18
			**HCN induced**	2.00 ± 0.00		7.00 ± 0.82	
	**R6-5**	175 µM	no induction	3.75 ± 0.50	1.15	13.75 ± 1.71	4.58
			**HCN induced**	3.25 ± 1.26		3.00 ± 0.00	
	**R6-8**	185 µM	no induction	6.75 ± 0.50	0.77	17.50 ± 1.73	3.33
			**HCN induced**	8.75 ± 0.50		5.25 ± 0.50	
HCN- strains	**DRY1-10**	0 µM	no induction	21.75 ± 2.50	3.95	11.50 ± 0.58	4.60
			**HCN induced**	5.50 ± 1.29		2.50 ± 0.58	
	**DRY4-5**	0 µM	no induction	19.25 ± 1.71	3.50	18.75 ± 1.89	8.33
			**HCN induced**	5.5 ± 1.29		2.25 ± 0.50	
	**RUM3-2**	0 µM	no induction	16.50 ± 0.58	3.47	15.00 ± 0.00	5.00
			**HCN induced**	4.75 ± 0.50		3.00 ± 0.00	
	**RUM3-9**	0 µM	no induction	25.00 ± 0.00	2.50	23.00 ± 1.41	23.00
			**HCN induced**	10.00 ± 1.15		1.00 ± 0.00	
	**K5-9**	0 µM	no induction	7.50 ± 0.58	3.00	5.75 ± 0.50	3.83
			**HCN induced**	2.50 ± 0.58		1.50 ± 0.58	
	**K7-8**	0 µM	no induction	25.00 ± 0.00	1.00	20.25 ± 0.50	2.03
			**HCN induced**	25.00 ± 0.00		10.00 ± 0.00	
	**K7-10**	0 µM	no induction	16.75 ± 0.96	0.67	25.00 ± 0.00	3.70
			**HCN induced**	25.00 ± 0.00		6.75 ± 0.50	
	**R2-1**	0 µM	no induction	7.00 ± 0.00	2.33	11.25 ± 0.50	3.46
			**HCN induced**	3.00 ± 0.00		3.25 ± 0.50	
	**R2-9**	0 µM	no induction	9.50 ± 0.58	0.66	16.25 ± 0.96	3.42
			**HCN induced**	14.50 ± 0.58		4.75 ± 0.50	

[1] The strain's potential to produce HCN at *in vitro* conditions. HCN is measured in liquid culture using growth medium supplemented with glycine.
[2] Inhibition of growth was examined on two types of test media, one supplemented with glycine (LBA_{gly}), which induced HCN production, and one without the supplement (LBA), on which HCN production was not additionally induced.
[3] Calculated inhibition, ratio between fungus colony diameter on both types of medium, with and without glycine supplement.

TABLE S2 | Comparison of dry mass of seed and young plant. Dry mass of seeds and 30 day old whole young plants growing on mineral substrate (data from subgroups growing on limestone and granite substrates were pooled together) was determined to calculate the mass increase factor after germination.

	French sorrel (*Rumex scutatus*)	maize (*Zea mays*)
seed dry mass (mg)	1.4 ± 0.2	258.6 ± 35.5
plant dry mass (mg)	7.7 ± 3.8	243.1 ± 76.1
**mass increase factor**	**5.5 ± 0.5**	**0.9 ± 0.3**
