# Supplementary material for: Hydrogen Cyanide in the Rhizosphere: Not Suppressing Plant Pathogens, but Rather Regulating Availability of Phosphate
Source: Front Microbiol. 2016 Nov 18;7:1785. doi: 10.3389/fmicb.2016.01785 (PMC5114478; doi:10.3389/fmicb.2016.01785)
Supplement: Supplementary file 2 [file Image1.pdf]

Hydrogen cyanide in the rhizosphere: not suppressing plant pathogens, but rather regulating availability of phosphate

Tomaž Rijavec[1,2] and Aleš Lapanje[1,2,3,*]

[1] Institute of Metagenomics and Microbial Technologies, Clevelandska ulica 19, 1000 Ljubljana, Slovenia

[2] Department of Environmental Sciences, Jožef Stefan Institute, Jamova cesta 39, 1000 Ljubljana, Slovenia

[3] Remote Controlled Theranostic Systems Lab, Saratov state university, Astrakhanskaya 83, Saratov, Russian Federation

Correspondence: Aleš Lapanje, Institute of Metagenomics and Microbial Technologies, Clevelandska ulica 19, 1000 Ljubljana, Slovenia, 00 386 68 604 979

SUPPLEMENTARY FIGURES

S1-S6

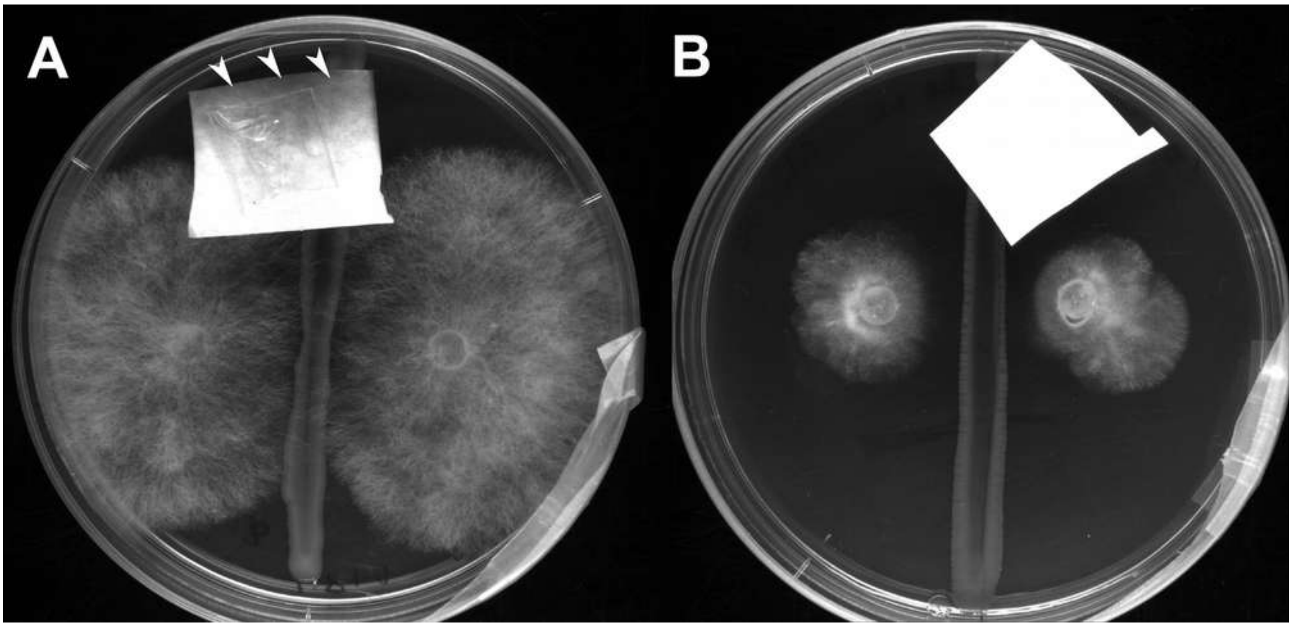

FIGURE S1 | Semi-quantitative test for HCN production by bacteria growing on petri dishes. When HCN production is present, the white indicator paper colors blue (represented in grayscale as dark black), as labeled by white arrowheads. Inhibition of growth of *Fusarium* spp. phytopathogens by HCN producing environmental isolates was tested by streaking the bacterial culture in the middle of the plate and by placing two agar plugs with fungal mycelium on both sides. HCN production was induced by adding glycine to the growth medium. (A) Example of HCN+ isolate (HCN reaction: positive) not exhibiting any inhibition of fungal growth. (B) Example of HCN- isoalte (HCN reaction: negative) exhibiting inhibition of fungal growth. Author of original photos: Sanja Sekuvanić, Msc.

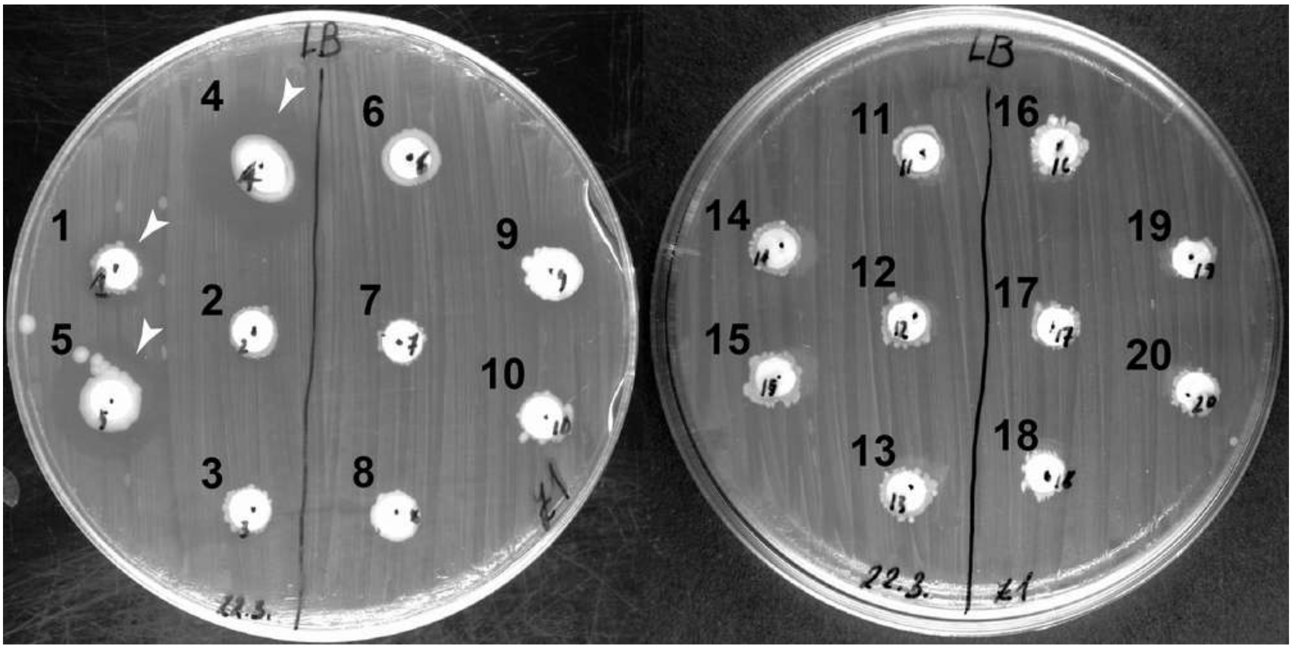

FIGURE | S2. Biocontrol assay of phytopathogenic *Pseudomonas syringae* pv. *syringae*. Growth inhibition of *P. syringae* on LBA medium can be observed as a clear zone surrounding the white paper disks (white arrow heads). Bacterial environmental isolates tested for biocontrol potential were applied to agar surface on paper disks (white circles). Strains: (1) DRY1-2, HCN+ (2) DRY9-8, HCN+ (3) RUM2-2, HCN+ (4) RUM10-10, HCN+ (5) CHA0, HCN+ (6) DRY1-10, HCN- (7) DRY4-5, HCN- (8) RUM5-1, HCN+ (9) RUM3-2, HCN- (10) RUM3-9, HCN- (11) K9-7, HCN+ (12) R6-5, HCN+ (13) R6-8, HCN+ (14) R7-1, HCN+ (15) R10-1, HCN+ (16) K5-9, HCN- (17) K7-8, HCN- (18) K7-10, HCN- (19) R2-1, HCN- (20) R2-9. HCN-.

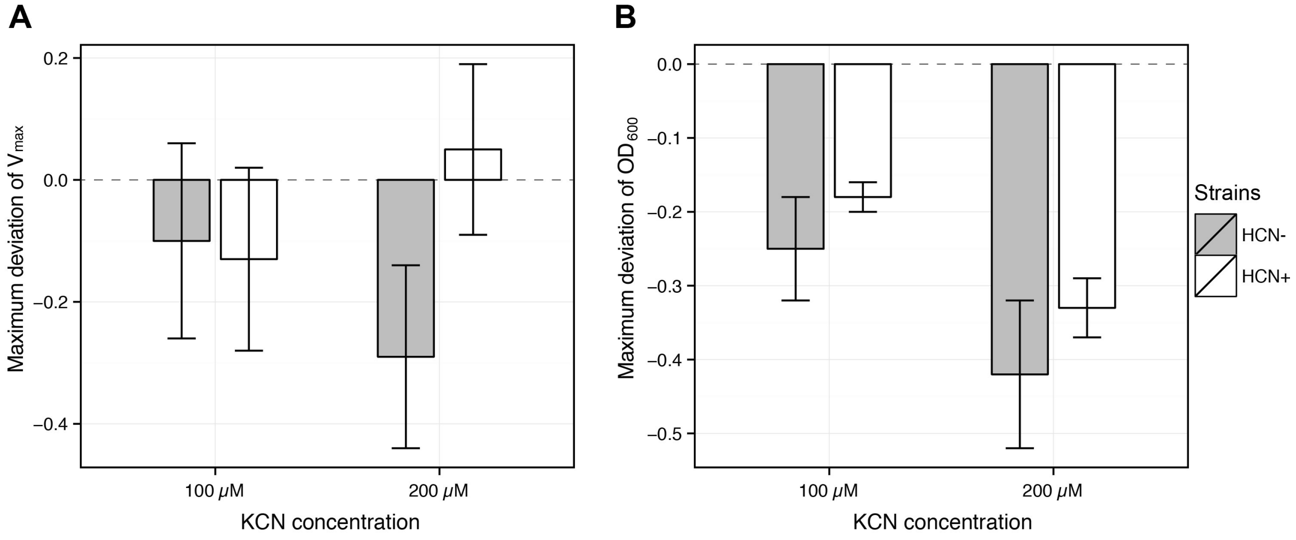

FIGURE S3 | Effect of cyanide on maximum deviation of V_{max} and OD_{600}. The growth curves of bacterial strains are affected by KCN supplemented to the growth medium (100 µM KCN, dark gray and 200 µM KCN, black). Control growth was assessed in medium without KCN. The pH of the medium (7.4) was unaltered after addition of KCN and during the growth of cultures. Data represent (A) the deviation of maximal growth rate, v_{max} and (B) maximal calculated deviations of OD_{600} from control growth (right) at 100 µM (dark gray) and 200 µM (black) KCN supplement. Average ± SE of 4 strains in each group and 4 replicate growth experiments for each strain is displayed.

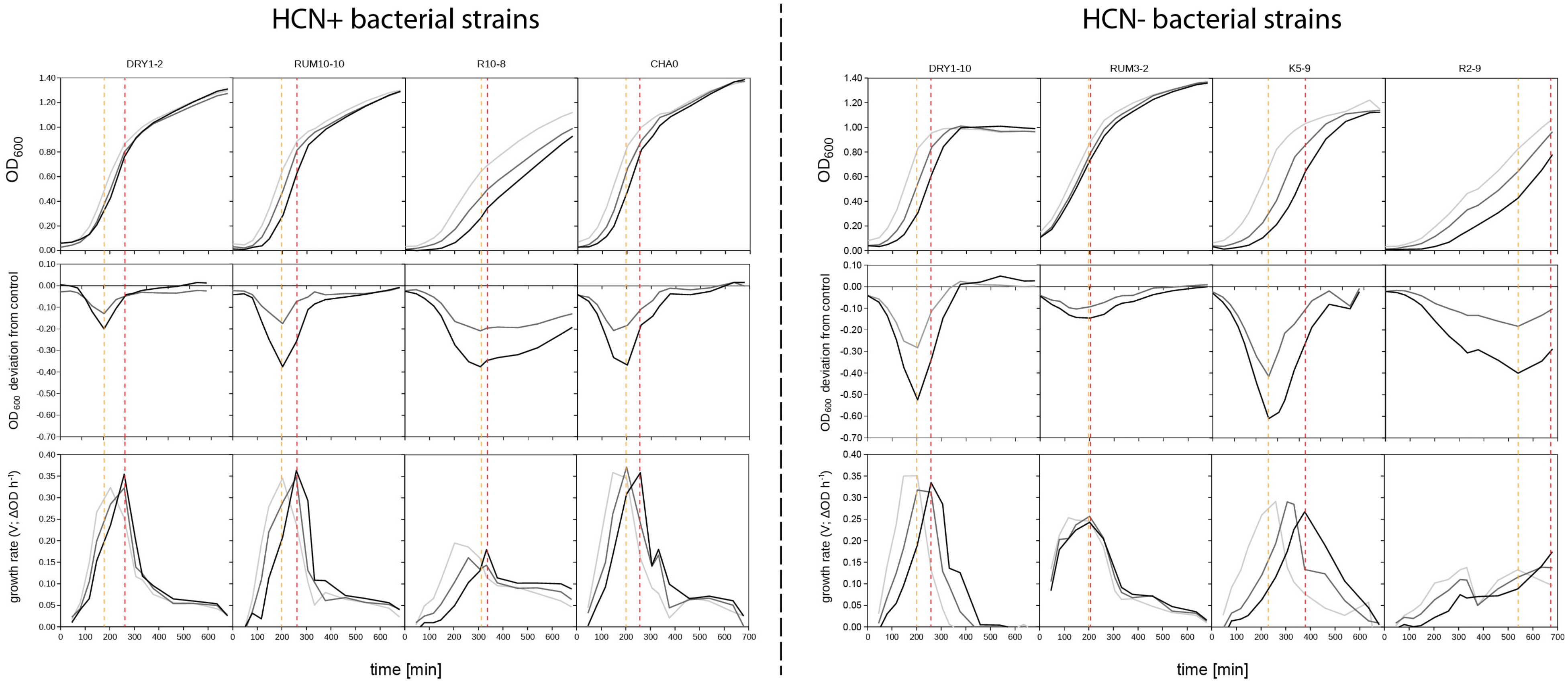

FIGURE S4 | Effect of KCN on growth kinetics of isolated bacteria. Growth kinetics of four HCN+ and four HCN- strains were determined in growth media supplemented with 200 and 100 μM KCN and without the KCN supplement (control group). The growth curve is represented as change of OD_{600} over time (top). Difference of curves is calculated by subtracting the growth curve obtained in the presence of KCN (200 or 100 μM) from curve of control group (center). Growth rate is calculated as $\Delta OD_{600}\ h^{-1}$ (bottom). Time of maximum OD_{600} deviation from control (orange dashed line), time of maximum growth rate of control (red dashed line).

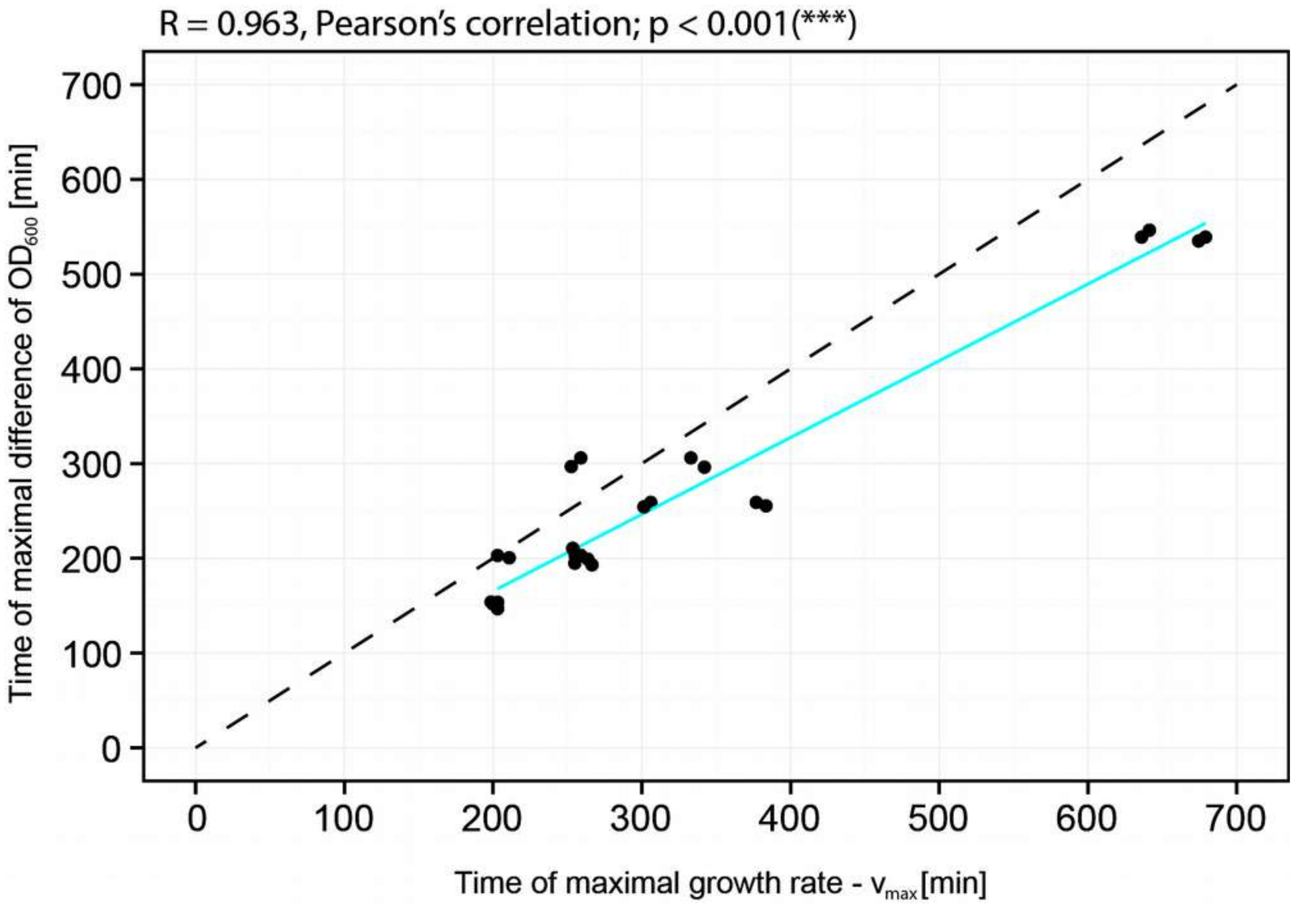

FIGURE S5 | Temporal correlation between maximal growth rate and maximal difference of OD_{600}. The blue line represents the linear regression line. The black dashed line represents complete temporal matching of both parameters.

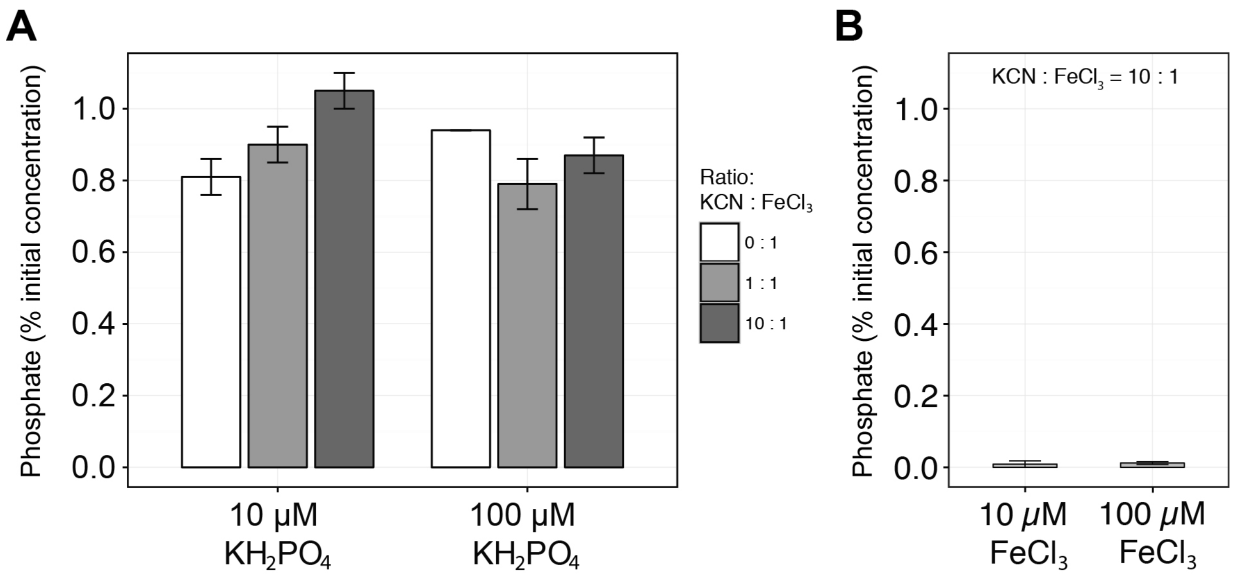

FIGURE S6 | Availability of PO_4^{3-} when KH_2PO_4 is mixed with $FeCl_3$ in a 1:1 ratio. (A) The mixture of $FeCl_3$ and KH_2PO_4 was prepared in a ratio of 1:1 using 10 and 100 µM KH_2PO_4. KCN was added afterwards in the final ratios $FeCl_3$:KCN = 1:1 and 1:10. (B) Negative control without KH_2PO_4, where only $FeCl_3$ and KCN were mixed in ratio 1:10. Signals of 10 and 100 µM KH_2PO_4 were used as 100% initial concentration. Data represent average ± SE (n = 3). pH of the solutions were: 2.9 (no KCN present), 3.9 ($FeCl_3$:KCN = 1:1) and 9.4 ($FeCl_3$:KCN = 1:10).
